# Kynurenine 3-Monooxygenase Interacts with Huntingtin at the Outer Mitochondrial Membrane

**DOI:** 10.3390/biomedicines10092294

**Published:** 2022-09-15

**Authors:** Aisha M. Swaih, Carlo Breda, Korrapati V. Sathyasaikumar, Natalie Allcock, Mary E. W. Collier, Robert P. Mason, Adam Feasby, Federico Herrera, Tiago F. Outeiro, Robert Schwarcz, Mariaelena Repici, Flaviano Giorgini

**Affiliations:** 1 Department of Genetics and Genome Biology, University of Leicester, Leicester LE1 7RH, UK; 2 Leicester School of Allied Health Sciences, Faculty of Health and Life Sciences, De Montfort University, Leicester LE1 9BH, UK; 3 Maryland Psychiatric Research Center, Department of Psychiatry, University of Maryland School of Medicine, Baltimore, MD 21201, USA; 4 Core Biotechnology Services, Adrian Building, University of Leicester, University Road, Leicester LE1 7RH, UK; 5 Cell Structure and Dynamics Laboratory, Department of Chemistry and Biochemistry, Faculty of Sciences, University of Lisbon, 1749-016 Lisbon, Portugal; 6 BioISI—Biosystems & Integrative Sciences Institute, Faculty of Sciences, University of Lisbon, 1749-016 Lisbon, Portugal; 7 Department of Experimental Neurodegeneration, Center for Biostructural Imaging of Neurodegeneration, University Medical Center Göttingen, 37073 Göttingen, Germany; 8 Max Planck Institute for Experimental Medicine, 37075 Göttingen, Germany; 9 Translational and Clinical Research Institute, Faculty of Medical Sciences, Newcastle University, Newcastle NE2 4HH, UK; 10 Scientific Employee with an Honorary Contract at German Center for Neurodegenerative Diseases (DZNE), 37075 Göttingen, Germany; 11 College of Health and Life Sciences, Aston University, Aston Triangle, Birmingham B4 7ET, UK

**Keywords:** kynurenine 3-monooxygenase, Huntington’s disease, huntingtin, mitochondria, BiFC, live cell imaging

## Abstract

The flavoprotein kynurenine 3-monooxygenase (KMO) is localised to the outer mitochondrial membrane and catalyses the synthesis of 3-hydroxykynurenine from L-kynurenine, a key step in the kynurenine pathway (KP) of tryptophan degradation. Perturbation of KP metabolism due to inflammation has long been associated with the pathogenesis of several neurodegenerative disorders, including Huntington’s disease (HD)—which is caused by the expansion of a polyglutamine stretch in the huntingtin (HTT) protein. While HTT is primarily localised to the cytoplasm, it also associates with mitochondria, where it may physically interact with KMO. In order to test this hypothesis, we employed bimolecular fluorescence complementation (BiFC) and found that KMO physically interacts with soluble HTT exon 1 protein fragment in living cells. Notably, expansion of the disease-causing polyglutamine tract in HTT leads to the formation of proteinaceous intracellular inclusions that disrupt this interaction with KMO, markedly decreasing BiFC efficiency. Using confocal microscopy and ultrastructural analysis, we determined KMO and HTT localisation within the cell and found that the KMO-HTT interaction is localized to the outer mitochondrial membrane. These data suggest that KMO may interact with a pool of HTT at the mitochondrial membrane, highlighting a possible physiological role for mitochondrial HTT. The KMO-HTT interaction is abrogated upon polyglutamine expansion, which may indicate a heretofore unrecognized relevance in the pathogenesis of this disorder.

## 1. Introduction

Kynurenine 3-monooxygenase (KMO) is a flavin monooxygenase that is primarily expressed in microglia within the central nervous system (CNS), although it has also been detected in neurons [1,2]. KMO lies at a key branchpoint of the kynurenine pathway (KP) of tryptophan degradation, where it catalyses the conversion of L-kynurenine to the neurotoxin 3-hydroxykynurenine (3-HK) [2]. Some KP metabolites have been shown to be neuroactive (e.g., 3-HK and the excitotoxin quinolinic acid), and it is hypothesised that imbalances in levels of these metabolites contribute to the pathogenesis of several neurodegenerative disorders, including Huntington’s disease (HD) [3]. HD is an autosomal dominantly inherited neurodegenerative disorder characterised by selective loss of medium spiny striatal neurons in the basal ganglia and the accumulation of protein aggregates within cells [4]. HD is caused by an unstable expansion in a glutamine encoding CAG tract within exon 1 of the *HTT* gene [5], which encodes the huntingtin (HTT) protein. This mutation leads to the expansion of a stretch of glutamine residues (polyQ) within the N-terminus of HTT, resulting in a toxic gain of function mechanism which is believed to “poison” several cellular processes [6,7]. The formation of N-terminal fragments of mutant HTT via caspase cleavage [8] and splicing defects [9] is associated with HD pathogenesis. Indeed, many models of the disorder are based on the expression of an N-terminal fragment of HTT encoded by the first exon of the gene (i.e., exon 1 fragment).

KMO activity is upregulated in several brain regions of HD model mice [10], and normalisation of KP metabolic imbalance via KMO inhibition ameliorates disease-relevant phenotypes in several models of HD and other neurodegenerative disorders [11,12,13,14,15,16]. However, though KP perturbations in the brain may play a role in the pathogenesis of HD, recent work found no changes in levels of several key KP metabolites in cerebrospinal fluid and plasma in pre-manifest and manifest HD individuals compared with healthy controls [17]. Nonetheless, recent work suggests that KMO inhibition may serve to dampen inflammation in microglia [18], and that ablation of the *Kmo* gene in HD model mice normalises peripheral inflammation [16]. This is of note, as both central and peripheral inflammation are observed in HD and may contribute to disease [19]. 

In addition to the KP, other cellular and metabolic perturbations have been implicated in HD pathogenesis, including defects in vesicle trafficking and mitochondrial dysfunction. Mitochondrial alterations have long been linked to HD, with defects associated with impaired energy metabolism and increased oxidative stress [20]. While HTT is predominantly cytoplasmic and membrane-associated [21,22], mitochondrial localisation has been observed [23,24]. Several recent studies have suggested that mitochondrial protein import is inhibited by mutant HTT, and this may be mediated by direct interactions with the protein translocase TIM23 [25,26,27]. Thus, a pool of mutant HTT may be localised to mitochondria, resulting in disruption of the normal functioning of this organelle and ultimately contributing to disease phenotypes. Notably, mitochondria are responsible for generating reactive oxygen species (ROS) and can respond to cellular changes induced by ROS production, acting as key players in neuroinflammation [28]. Indeed, the relationship between mitochondria and the immune response contributes to several inflammatory diseases, including neurodegenerative disorders.

KMO has been found to localise solely to the outer mitochondrial membrane [29,30]. We were thus intrigued by the possibility that KMO and HTT may interact at the outer mitochondrial membrane, and that this interaction might by perturbed in HD. To investigate this possibility, we employed the bimolecular fluorescence complementation (BiFC) assay [31] to study the interactions of KMO with both wild type (WT) and mutant exon 1 fragments of HTT in living cells. We found that KMO interacts with WT HTT (HTT19Q), while this interaction was abrogated with a mutant HTT construct (HTT97Q). Extended confocal and transmission electron microscopy (TEM) studies revealed that the KMO-HTT complexes indeed localised to the outer mitochondrial membrane. Notably, the reduced KMO-HTT interactions observed due to polyQ expansion correlated with an increased propensity of the HTT constructs to form cytoplasmic aggregates, suggesting that aggregation sequesters HTT away from normal interactions with KMO. 

## 2. Methods

### 2.1. Construction of KMO and HTT Plasmids

Full length human KMO (flKMO) cDNA was obtained from Origene (TP322594) (GenBank accession: NM_003679), and truncated KMO (tKMO) cDNA was sourced from Gene Service (clone 3934714). tKMO resembles the KMO splice isoform 2 with a C-terminal truncation, resulting in the deletion of the second transmembrane domain. The flKMO cDNA had a single nucleotide polymorphism at position 786 CC**A** which was reversed back to CC**G** using site-directed mutagenesis. Single base pair substitution was achieved by amplifying human flKMO R452C using primers that introduced the mutagenesis in the newly synthesized DNA. The forward primer (GTCACCACGATCTTTCCTCCGCTTGAGAAGACCATGGAAC), and the reverse primer (GTTCCATGGTCTTCTCAAGCGGAGGAAAGATCGTGGTGAC) incorporating the mutation were designed using PrimerX online software. All PCR products were digested overnight with *DpnI* to remove methylated template DNA before being transformed into chemically competent *Escherichia coli* strain DH5α. Mutagenesis was confirmed by DNA sequencing. Cloning of all the constructs was mediated by CloneJET PCR Cloning Kit (Thermo Fisher Scientific, Loughborough, UK). All primers that were used are listed in Table S1. RFP-tagged constructs: amplified flKMO and tKMO cDNA were cloned into *KpnI* sites of pcDNA3.1 mammalian expression vector containing RFP. Untagged flKMO and MYC were amplified and cloned into *KpnI* and *PstI* sites of pcDNA3.1. KMO BiFC constructs: the PCR products of flKMO and tKMO were cloned into *NheI* and *XhoI* sites of pcDNA3.1 encoding C-terminal of CFP (CC). Generation of the correct constructs was validated by DNA sequencing. We employed our previously characterised VC and VN tagged HTT exon 1 BiFC constructs [32] (19Q-VN, 25Q-VC, 97Q-VN, 97Q-VC; CAG repeat length confirmed by DNA sequencing) and also generated the 46Q-VN construct as described [32]. The HTT25Q-GFP construct utilised (Figure 7D) has been previously described [33]. 

### 2.2. Mammalian Cell Culture Growth Conditions and Transfection

HEK293T cells were routinely cultured in Dulbecco’s modified Eagle medium (DMEM) (GlutaMAX, Thermo Fisher Scientific, Loughborough, UK) supplemented with 10% fetal bovine serum (FBS, Thermo Fisher Scientific, Loughborough, UK), 1000 units mL^−1^ penicillin and 100 µg·mL^−1^ streptomycin. Cells were incubated in a humidified incubator at 37 °C with 5% CO_2_. HEK293T cells were seeded at the required density on 0.01% Poly-Lysine pre-coated plates/dishes depending on the experiment, and transiently transfected, for 24, 48 or 72 h, using Effectene Transfection Reagent (Qiagen, Hilden, Germany) according to the manufacturer’s instructions.

### 2.3. Immunocytochemistry (ICC) 

HEK293T cells were seeded on 20 mm sterile coverslips in a 6-well plate at a density of 1.5 × 10^5^ cells/well. For live cell imaging, cells were seeded into 35 mm ibiTreat dishes (Ibidi) at a density of 1 × 10^5^ cells/dish. After 24–48 h, cells were fixed in 4% paraformaldehyde (PFA), as previously described [34]. MitoTracker CMXRox (M-7512, Thermo Fisher Scientific, Loughborough, UK) was used as a mitochondrial marker. Cells were stained with 100 nM MitoTracker CMXRox for 30 min and washed once with phosphate-buffered saline (PBS) prior to fixation. ICC on stained cells was performed as follows: after fixation, cells were permeabilised with 0.2% Triton in PBS for 10 min. Cells were then washed three times in 0.1% Tween 20 in PBS (PBS-T). Blocking and antibody incubation steps were carried out in 1% BSA in PBS-T, and all washing steps used PBS-T. Primary antibodies and dilutions are listed in Table S2. Secondary antibodies, Alexa Fluor 488, 546, 555 or 647 (Invitrogen, Inchinnan, UK), were diluted 1:500. Morphological analysis was performed by confocal microscopy (Olympus FV1000, Tokyo, Japan). Confocal images were deconvolved using Essential Huygens software, and microscopic parameters were set as follows: for Hoechst, excitation at 405 nm and emission 422 nm; for Alexa 488, excitation at 488 nm and emission at 508; for RFP, excitation at 559 nm and emission at 608 nm; for Alexa 555, excitation at 555 nm and emission at 568 nm; and for Alexa 647, excitation at 647 nm and emission at 670 nm. Confocal studies coupled with ICCB analyses were used to analyse some of the acquired images. The ICCB analyses were facilitated by the ImageJ (Version 1.50d) plugin JACoP (Just Another Co-localisation Plugin, https://imagej.net/JaCoP (access on 29 August 2022)). JACoP groups the main ICCB tools and permits the use of various methods, such as Pearson’s coefficient (PC) and Mander’s coefficient (M1 and M2) based analyses [35]. 

### 2.4. Transmission Electron Microscopy 

HEK293T cells were seeded on 9 mm coverslips in a 12-well plate at a density of 1 × 10^5^ cells/well. After 48 h, cells were fixed in 4% PFA + 0.05% glutaraldehyde (GA) in PBS for 20 min at room temperature. Fixed cells were washed three times in PBS for 15 min followed by four dehydration steps in ethanol: 30%, 50%, 70% and finally 90% (each for 30 min at 4 °C). Three further dehydration steps were applied in a solution of 90% ethanol: LR White resin (Agar Scientific Ltd., Stansted, UK). Ratios were 2:1, 1:1 and 1:2, each used for 30 min at 4 °C. Finally, cells were incubated in 100% LR White resin for 30 min at 4 °C and then stored in 100% LR white at 4 °C. Fresh LR White resin was added three times for 2 h at 4 °C. Samples embedded in LR White resin were polymerised for 16 h under UV light at 4 °C and in a N_2_ atmosphere. Polymerised samples were then processed into thin sections of 90 nm using an Ultracut S Ultramicrotome (Leica, Wetzler, Germany) and collected onto 200 squares gold mesh grids for immunolabelling. Grid-mounted sections were blocked in 30 µL of 1% BSA in 0.1% Tween 20 PBS (PBS-T) for 30 min. Grids were then incubated with primary antibody (Table S2) in 1% BSA in PBS-T for 2 h, followed by five 3 min washes with 1% BSA in PBS-T and, subsequently, 1.5 h incubation with the appropriate IgG (anti-mouse or anti-rabbit) gold conjugate secondary antibody (BBI Solutions, 1:100) in 1% BSA in PBS-T. Grids were then washed once for 3 min with 1% BSA in PBS-T followed by four 2-min washes with distilled de-ionised water. Finally, grids were counter-stained in 2% uranyl acetate (Agar Scientific Ltd., Stansted, UK) for 10 min. Cells were visualised on a JEOL JEM-1400 TEM (JEOL Ltd., Tokyo, Japan) with an accelerating voltage of 80 kV from 5–10 grid squares, and images were captured using a Megaview III digital camera with iTEM software. 

### 2.5. Bimolecular Fluorescence Complementation (BiFC) Assay

HEK293T cells were seeded on a 6-well plate as before. At 48 h post transfection, media was replaced with phenol red-free DMEM media supplemented with 10% FBS, 2 mM L-glutamine, 1000 units mL^−1^ penicillin and 100 µg·mL^−1^ streptomycin. Live cell imaging was performed at 37 °C and 5% CO_2_ using an Olympus ScanR screening station. Detection of the GFP signal was at 492/18 nm excitation filter and 535/50 nm emission filter, whereas RFP detection was at 556/30 nm excitation filter and emission filter of 590–650 nm. One hundred images were taken per well and analysed using ScanR analysis software. The software identifies red cells, measures the area of each cell by detecting the extent of the RFP signal, and quantifies the total intensity of the BiFC signal (green). The ratio between the total green intensity and the cell area, expressed as mean green signal intensity, was used for normalising the BiFC assay. 

### 2.6. Immunoblotting and Filter Trap Assay

Lysis of HEK293T cells and immunoblotting were performed as previously described [34]. Primary antibodies and their dilutions are listed in Table S2. Secondary antibodies anti-rabbit/anti-mouse IgGs, peroxidase conjugated (Vector Laboratories, Peterborough, UK) were diluted 1:10,000. For filter trap, HEK293T cells were seeded on a 6-well plate as before. After 48 h of transfection with HTT constructs, cells were lysed in CellLytic (MT Cell Lysis Reagent, Sigma Aldrich, Dorset, UK) supplemented with 1X Roche cocktail protease inhibitors. Subsequently, lysates were left for 15 min on a platform rocker at room temperature. Then, 250 U/mL of Benzonase (Pierce Universal Nuclease, Thermo Fisher Scientific, Loughborough, UK) was added to the lysates, followed by incubation on ice for 30 min. Protein concentration of the lysates was determined using an Implen NanoPhotometer. Lysates were diluted in PBS containing 2% (*w*/*v*) SDS and 50 mM DTT to 1.5 μg/μL, and denatured at 98 °C for 3 min. Cellulose acetate (aggregate binding) membrane was equilibrated in 0.1% (*w*/*v*) SDS in PBS, and used for filtering 150 μL of freshly diluted lysates (50 µg and 100 µg). The membrane was then blocked in 3% (*w*/*v*) milk in 1X TBS-T at 4 °C overnight. Incubation with anti-GFP primary antibody (1:10,000, Abcam) was performed for 1 h at room temperature. After three washes in 1X TBS-T for 10 min, the membrane was incubated for 1 h at room temperature with the anti-rabbit IgG, peroxidase conjugated secondary antibody (Vector Laboratories, Peterborough, UK; 1:20,000 dilution in 1X TBS-T). This was followed by three washes in 1X TBS-T for 10 min. The membrane was then developed using Enhanced Chemiluminescence (ECL), SuperSignal West Dura (Thermo Fisher Scientific, Loughborough, UK) for 5 min. 

### 2.7. Kynurenine 3-Monooxygenase Activity

Cell pellets were thawed and homogenized by sonication in 400 µL of ultrapure water (Branson Ultrasonics, Brookfield, CT, USA). The homogenate was further diluted (1:2, *v*/*v*) in 100 mM Tris-HCl (pH 8.1) containing 10 mM KCl and 1 mM EDTA. Then, 80 µL of the preparation was incubated for 40 min at 37 °C in a solution containing 1 mM NADPH, 3 mM glucose-6-phosphate, 1 U/mL glucose-6 phosphate dehydrogenase, 100 µM L-kynurenine, 10 mM KCl and 1 mM EDTA, in a total volume of 200 µL. Blanks were obtained by including the specific enzyme inhibitor Ro 61-8048 (100 μM) in the incubation solution. The reaction was stopped by the addition of 50 μL of 6% perchloric acid. The samples were centrifuged at 16,000× *g*, 15 min, and the resulting supernatants were diluted as needed. Then, 20 μL of the solution was applied to a 3 μm HPLC column (HR-80; 80 mm × 4.6 mm, ESA), using a mobile phase consisting of 1.5% acetonitrile, 0.9% triethylamine, 0.59% phosphoric acid, 0.27 mM EDTA and 8.9 mM sodium heptane sulfonic acid. In the eluate, the reaction product, 3-HK, was detected electrochemically using an Eicom HTEC 500 detector (oxidation potential: +0.5 V; flow rate: 0.5 mL/min). The retention time of 3-HK was ~11 min. Protein was determined according to Lowry et al. [36], using bovine serum albumin as a standard.

### 2.8. MYC-Trap Immunoprecipitation 

HEK293T cells were grown on 10 cm Petri dishes at a density of 2 × 10^6^ cells/dish and transfected as above with 1 µg of either 1–90 HTT-Q23-MYC, 1–90 HTT-Q145-MYC or MYC and with 1 µg of untagged flKMO. After 48 h, cells were washed in ice-cold PBS and treated with 2 mM dithiobis (succinimidyl propionate) (DSP, Thermo Fisher Scientific, Loughborough, UK) in PBS at room temperature for 30 min. Next, 20 mM of Tris-HCl pH 7.4 was used for blocking DSP. The cells were left for 10 min at room temperature and then washed twice with PBS. Cells were lysed as described above, and immunoprecipitation was performed following the MYC-Trap Magnetic Agarose protocol provided by the manufacturer (Chromotek, Manchester, UK).

### 2.9. RFP-Trap Immunoprecipitation

HEK293T cells were grown in T75 flasks 2 × 10^6^ cells/flask and transfected with either 15 µg of flKMO-RFP or RFP using Lipofectamine 3000 (Thermo Fisher) for 48 h. Cells were washed twice with ice-cold PBS and lysed for 30 min in 1 mL of ice-cold lysis buffer (10 mM Tris-HCl, pH 7.5, 150 mM NaCl, 0.5 mM EDTA, 0.05 % n-dodecyl β-D-maltoside, 1% (*v*/*v*) HALT protease inhibitor cocktail, 1% (*v*/*v*) HALT phosphatase inhibitor cocktail (Thermo Fisher Scientific, Loughborough, UK). Four aliquots of each sample (250 µL) were diluted 1:1 with dilution buffer (10 mM Tris-HCl, pH 7.5, 150 mM NaCl, 0.5 mM EDTA, 1% (*v*/*v*) HALT protease inhibitor cocktail, 1% (*v*/*v*) HALT phosphatase inhibitor cocktail) and lysates were cleared with Binding Control Agarose (20 µL per aliquot) (Chromotek, Manchester, UK) for 30 min at 4 °C with rotation. The supernatant was then incubated with 25 µL of RFP-Trap Agarose (Chromotek, Manchester, UK) for 1 h at 4 °C with rotation. The agarose beads were washed thrice with 500 µL wash buffer (10 mM Tris-HCl, pH 7.5, 150 mM NaCl, 0.5 mM EDTA). Proteins were eluted in 30 µL 2× SDS-PAGE loading buffer and were separated by 10% SDS-PAGE and then transferred onto nitrocellulose membranes. The membranes were incubated with HTT antibody followed by an anti-mouse HRP conjugated secondary antibody (1:5000; Vector Laboratories, Peterborough, UK). 

### 2.10. Statistical Analysis

All statistical analyses were carried out as described in the figure legends using GraphPad Prism 6 (Version 6.02), and all data were expressed as the mean ± SEM.

## 3. Results

### 3.1. Human KMO Localises to the Outer Mitochondrial Membrane in HEK293T Cells

**Figure 1:**
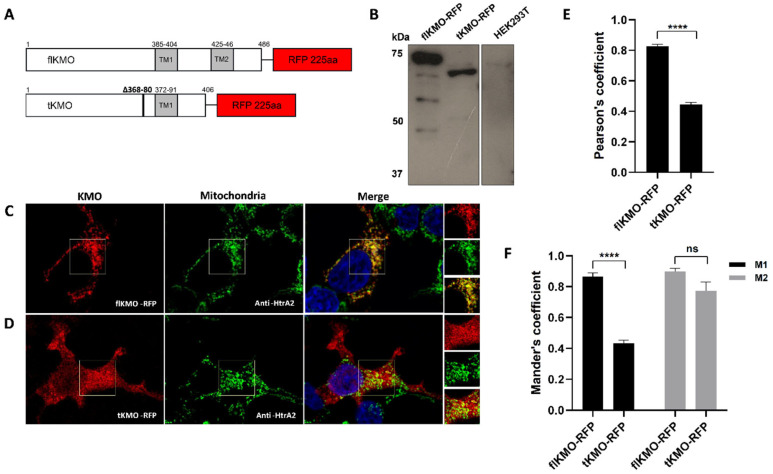
**Exogenous KMO expression and localisation in HEK293T cells.** (**A**) Schematic representation of KMO ICC constructs: flKMO-RFP and tKMO-RFP. Grey boxes show putative transmembrane domains (TM). tKMO has a deletion of amino acids from position 368 to 380 that resembles the deletion in KMO isoform 2, but is also truncated at its C-terminus, with 67 aa missing from the protein. (**B**) Immunoblotting of KMO constructs using anti-KMO antibody (10698-1-AP). (**C**,**D**) HEK293T cells were transfected with the flKMO-RFP (**C**), Left panel)) or tKMO-RFP ((**D**), Left panel) constructs and fixed 24 h after transfection; ((**C**,**D**) middle panels): immunolabelling for the mitochondrial protein HtrA2 using anti-HtrA2 antibody (AF1458) (Alexa Fluor 488); ((**C**,**D**) right panels): merge of the RFP and anti HtrA2 signal. Nuclei were stained with Hoechst 33342. Scale bar = 8 µm. flKMO-RFP depicts mitochondrial localisation (punctate structures), whereas the tKMO-RFP signal is diffuse throughout the cell. The squares on the images indicate the selected areas for co-localisation analysis (an enlarged view of the selected area is shown on the side of each merge panel). (**E**,**F**) Co-localisation analysis of optical z-sections from eight deconvolved confocal images, using JACoP in ImageJ. (**E**) Pearson’s coefficient shows a significant difference in the mitochondrial co-localisation of flKMO-RFP and tKMO-RFP. (**F**) Mander’s coefficient correlation. M1 represents the red signal overlapping the green signal, while M2 indicates the green signal overlapping the red signal. **** (*p* < 0.0001), for unpaired *t*-test. ns = not significant. Data are expressed as mean ± SEM (*n* = 8).

To investigate the cellular localisation of human KMO, RFP-tagged constructs were generated and transfected into HEK293T cells, and KMO expression was confirmed by immunoblotting (Figure 1A,B). Cellular localisation of KMO was determined by confocal microscopy coupled with immunofluorescence. As expected, full length KMO tagged with RFP (flKMO-RFP) had a punctate signal suggesting mitochondrial localisation, while a truncated version of KMO (tKMO-RFP), which lacks a putative transmembrane domain, was found diffusely throughout the cell (Figure 1C,D). These cells were also immunolabelled for the mitochondrial marker HtrA2 to examine the mitochondrial localisation of the flKMO-RFP and tKMO-RFP signals. Whereas the flKMO-RFP signal overlayed almost perfectly with the mitochondrial green signal (Figure 1C), the tKMO-RFP signal was diffuse in the cytosol, with some evidence of mitochondrial and nuclear localisation (signal overlapping with mitochondrial or Hoechst staining, respectively) (Figure 1D). 

Intensity correlation coefficient-based (ICCB) analyses were used to evaluate the degree of co-localisation of the fluorescently tagged proteins with the mitochondria. Pearson’s coefficient (PC) and Mander’s coefficient (M1 and M2) analyses revealed strong co-localisation of flKMO-RFP and the mitochondrial marker (PC = 0.83 ± 0.013; M1 = 0.87 ± 0.023 and M2 = 0.90 ± 0.021; Figure 1E,F), while tKMO-RFP presented poor mitochondrial co-localisation (PCC = 0.44 ± 0.015; M1 = 0.43 ± 0.021 and M2 = 0.77 ± 0.057) (Figure 1E,F). These data suggest that the C-terminus is essential for mitochondrial targeting, and that a deletion of the putative second transmembrane domain leads to mislocalisation of the human KMO, as has been observed for pig liver KMO [29].

### 3.2. KMO Interacts with Soluble HTT in a PolyQ Dependent Manner at the Mitochondria 

**Figure 2:**
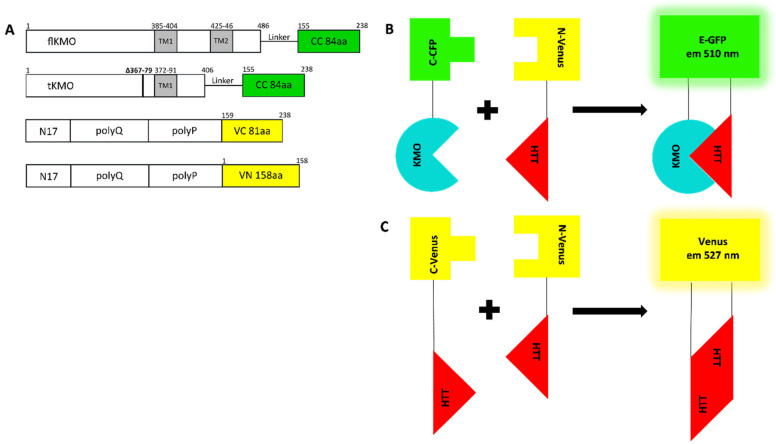
**Schematic representation of BiFC models and their complementation pairs.** (**A**) Schematic outline of the KMO and HTT BiFC constructs. flKMO/tKMO were fused C-terminally to N-terminus of CFP (CC) via (GGGGS)_2_ linker. The HTT BiFC constructs were composed of the N17, polyglutamine (polyQ) and polyproline (polyP) domains fused to either C-terminus or N-terminus halves of Venus (VC or VN). For each of the -VN or -VC HTT BiFC constructs in the illustration there are versions with different polyQ lengths: 25Q-VC, 19Q-VN, 46Q-VN, 97Q-VC and 97Q-VN. (**B**,**C**) Illustrations of BiFC combination pairs: (**B**) KMO and HTT pair that when interacting bring CC and VN together to form a fluorescence protein with 510 nm emission as in enhanced-GFP (E-GFP); (**C**) HTT pairs upon interactions, the two halves of Venus (VN and VC) re-constitute with emission 527 nm.

To study the interaction between KMO and HTT, we employed the BiFC assay, which is based on the reconstitution of nonfluorescent fragments of a fluorescent protein to study protein–protein interactions [31]. In our specific context, both KMO and HTT were fused to half of a fluorescent protein: if KMO interacts with HTT in living cells, or if HTT self-associates, the two nonfluorescent fragments are brought together, re-associate, and refold into a fluorescent complex (Figure 2A–C). HEK293T cells were employed as a general cell model to specifically address the question of these potential physical interactions.

**Figure 3:**
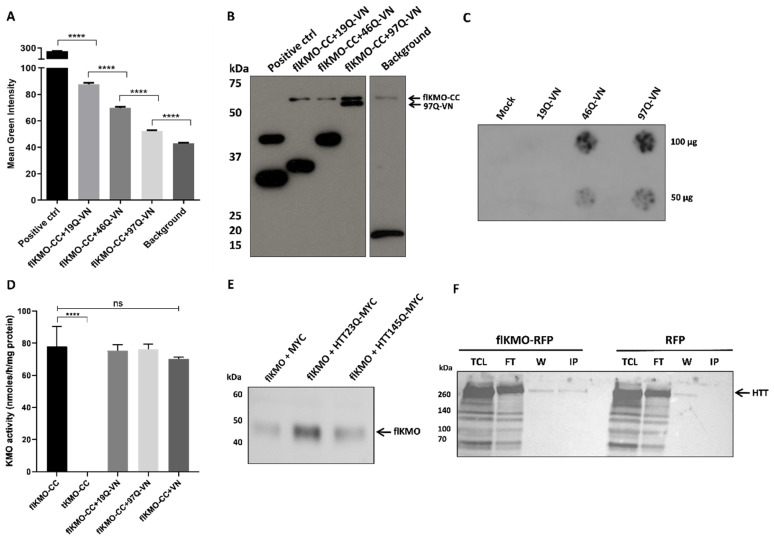
**Interaction of flKMO-CC and HTT-VN in HEK293T cells.** (**A**) Cells were transfected for 48 h with 0.16 µg of each plasmid and 0.08 µg of RFP. Fluorescence intensities were analysed using ScanR analysis software. Mean green intensity of the fluorescence complementation signal of three independent experiments shows a clear interaction between flKMO-CC and HTT-VN. The histogram shows a significant reduction in the fluorescence complementation as the polyQ length increases, which is significantly different from the background (background = flKMO-CC + VN-backbone); positive control = DJ-1-GN + DJ-1-CC from [34]. **** *p* < 0.0001, for one-way ANOVA, followed by Tukey’s multiple comparison tests. Data are expressed as mean ± SEM. The number of analysed cells ranged from 15,000 to 18,500 cells per condition. (**B**) A representative immunoblot of the lysates from one BiFC experiment shows the expression levels of flKMO-CC and the soluble fraction of HTT-VN proteins, using anti-GFP antibody (ab6556). Positive control = DJ-1-GN + DJ-1-CC. (**C**) Filter trap of cells lysates expressing only HTT-VN to reveal polyQ dependent protein aggregation, using anti-GFP antibody (ab6556; 1:10,000); each lysate was blotted in duplicate. (**D**) Activity of BiFC KMO constructs after expression in HEK293T cells: truncation leads to complete loss of activity, but flKMO-CC activity is maintained when co-expressed with BiFC-VN constructs. **** *p* < 0.0001 and ns = not significant for one-way ANOVA, followed by Tukey’s multiple comparison tests. Data are expressed as mean ± SEM. (**E**) HEK293T cells were transfected with untagged flKMO and either MYC alone, 1–90 amino acid HTT 23Q-MYC or 145Q-MYC constructs. Upon crosslinking, HTT constructs were pulled down by using MYC-Trap and revealed with anti-KMO (Proteintech, 1:1000). A strong interaction is observed between flKMO and 1–90 HTT-Q23-MYC whereas flKMO and 1–90 HTT-Q145-MYC displays a weaker interaction. (**F**) HEK293T cells were transfected with a construct expressing flKMO-RFP, which was pulled down with the RFP-Trap system and revealed with anti-HTT (4C8) antibody (MAB2166; 1:1000). An interaction between flKMO-RFP and endogenous HTT was detected. TCL = total cell lysate, FT = flow through, W = wash and IP = immunoprecipitation.

flKMO or tKMO were fused to the C-terminus of CFP (flKMO-CC and tKMO-CC, respectively) (Figure 2A) and, as expected, flKMO-CC was found to localise to mitochondria (Figure S1). For HTT, we used previously validated HTT BiFC constructs where different HTT exon 1 fragments (19Q, 46Q and 97Q) were fused at the C-termini to either half of Venus (C-terminus or N-terminus) [32] (Figure 3A). HEK293T cells were transfected with both BiFC constructs, along with a plasmid expressing RFP as an internal control for transfection and to allow for normalisation of the BiFC signal. The average fluorescence complementation of the KMO-HTT BiFC signals revealed that KMO and HTT specifically interact in living cells, and this interaction decreases as the polyQ length increases (Figure 3A), with a ~50% reduction in the interaction of flKMO-97Q complex in comparison to flKMO-19Q. Notably, despite the decreased interaction with increased polyQ length, flKMO-CC interaction with 97Q-VN was still above background levels (flKMO-CC + VN-backbone). 

After live imaging, cells were lysed, and immunoblotting was performed on all lysates to confirm protein expression of the BiFC constructs (Figure 3B). flKMO-CC was expressed in all experimental conditions, and HTT97Q-VN was clearly present at lower levels than the other HTT constructs (Figure 3B). This could be due to the majority of HTT97Q-VN being present in SDS-insoluble aggregates in cell extracts, thus being reduced in the soluble protein fractions tested. To address this issue, we next used a filter trap assay to detect insoluble, aggregated proteins. This experiment revealed robust aggregation of HTT97Q-VN, intermediate aggregation of HTT46Q-VN, and no aggregation of HTT19Q-VN (Figure 3C)—supporting the notion that the decreased levels of KMO-HTT97Q interactions observed are due to a lesser availability of soluble HTT97Q-VN. 

Interestingly, flKMO-CC activity was unaffected by co-expression with either HTT9Q-VN or HTT97Q-VN in HEK293T cells, suggesting that KMO-HTT interactions do not directly modulate enzymatic function. However, C-terminal truncation of KMO (tKMO-CC) led to complete loss of KMO activity (Figure 3D) and impaired its interaction with HTT (Figure S2). Notably, although KMO activity is elevated in the brain of R6/2 HD model mice [10], we did not see a similar effect here, likely due to neuroinflammation-dependent upregulation of KMO expression in vivo [37] and that the KMO BiFC constructs employed are not under transcriptional control of the endogenous *KMO* promoter. 

To confirm KMO-HTT interaction independently from the reconstitution of a fluorescent protein, we next used a biochemical approach. We transfected MYC-tagged WT and mutant HTT exon 1 constructs (HTT23Q-MYC and HTT145Q-MYC) into HEK293T cells along with an untagged flKMO construct. MYC-Trap co-immunoprecipitation of the protein complexes revealed a specific interaction between HTT23Q-MYC and flKMO (Figure 3E and Figure S3), which was robustly reduced with the polyglutamine expanded HTT145Q-MYC construct—confirming the BiFC results. In addition, we transfected the flKMO-RFP construct into HEK293T cells to further confirm potential KMO-HTT interactions. Notably, when flKMO-RFP was pulled down using the RFP-trap system, endogenous HTT was co-immunoprecipitated (Figure 3F and Figure S4). 

**Figure 4:**
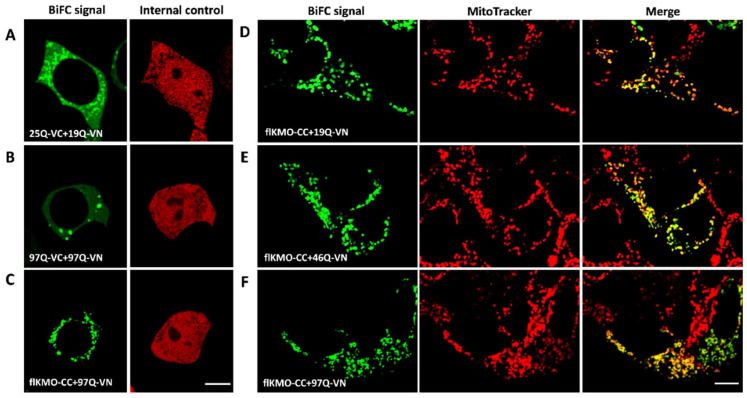
**Cellular localisation of BiFC complexes in live HEK293T cells, using confocal microscopy.** Cells were seeded on ibiTreat dishes and co-transfected with BiFC constructs for 48 h. (**A**–**C**) illustrate the HTT signal using the BiFC system (BiFC signal = left image, and internal control RFP signal = right image) in each panel. (**A**) Cells were transfected with 19Q-VN, 25Q-VC and RFP, the BiFC signal is cytosolic and slightly punctate. (**B**) Cells were transfected with 97Q pair and RFP; the BiFC signal is generally cytosolic, with HTT inclusions present. (**C**) Cells were transfected with flKMO-CC, 97Q-VN and RFP; the BiFC signal is mainly mitochondrial as suggested by the dotted appearance of the signal. Scale bar = 8 µm. (**D**–**F**) Localisation of KMO-BiFC complexes. Left panels show BiFC signal of the following pairs: (**D**) flKMO-CC and HTT19Q-VN, (**E**) flKMO-CC and HTT46Q-VN, and (**F**) flKMO-CC and HTT97Q-VN. Second column of panels (**D**–**F**): mitochondria stained with MitoTracker Red CMXRox (M-7512). Third column of panels (**D**–**F**): merge of the BiFC signal and the MitoTracker signal. Scale bar = 8 µm. The BiFC signal in panels (**D**–**F**) exhibits dotted structures that co-localise with the MitoTracker signal, as seen in the merge images in the right panels of (**D**–**F**). This confirms the mitochondrial localisation of all the BiFC complexes of flKMO-CC with different polyQ lengths of HTT-VN.

Lastly, to gain insight into the subcellular localisation of the HTT and KMO interaction, we performed BiFC (as described above) in living cells in combination with MitoTracker labelling to identify mitochondria. Both HTT19Q-VN and HTT46Q-VN generated a BiFC signal with flKMO-CC that co-localised with mitochondria (Figure 4D,E), indicating that the HTT-KMO interactions observed occur at the mitochondria. Interestingly, and not seen with the HTT97Q-BiFC pair (Figure 4A,B), interaction between flKMO-CC and 97Q-VN led to a punctate signal co-localising with mitochondria with no inclusions observed (Figure 4C,F). This indicates that flKMO-CC interacts with the soluble HTT-VN and is not sequestered into HTT aggregates. These data suggest that a soluble population of HTT97Q-VN is present and available to interact with KMO localised to the mitochondria (also confirmed in fixed HEK293T cells, Figure S5F). Indeed, immunolabelling of endogenous HTT in HEK293T revealed a subset of HTT localised to mitochondria which likely facilitate its interaction with KMO (Figures S6 and S7). 

### 3.3. HTT-VN Localises to Mitochondria in HEK293T Cells

**Figure 5:**
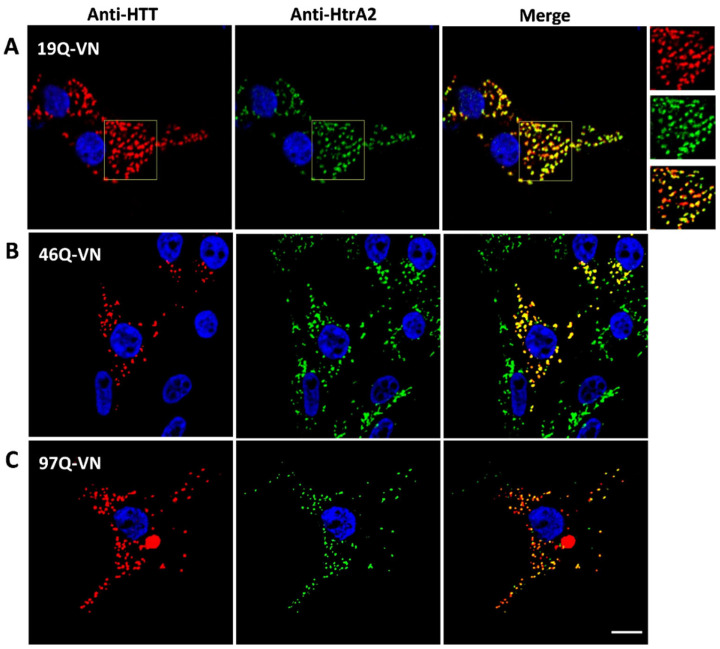
**Subcellular localisation of HTT-VN constructs.** HEK293T cells were transfected for 48 h with either 19Q-VN, 46Q-VN or 97Q-VN, then fixed and immunolabelled. (**A**) Co-localisation analysis of 19Q-VN and mitochondrial fluorescent signals on deconvolved confocal optical z-sections, using JACoP plugin in ImageJ. Left panel, 19Q-VN immunolabelling of anti-HTT (mEM48) antibody (MAB5374), (Alexa Fluor 647). Middle panel, mitochondrial immunolabelling of anti-HtrA2/Omi antibody (AF1458) (Alexa Fluor 555). Right panels: merge image of the HTT and mitochondrial signals. Nuclei were stained with Hoechst 33342. Scale bar = 8 µm. Co-localisation analyses were carried out on the regions of interest indicated on the images, and an enlarged image is presented on the right of panel (A). The 19Q-VN signal appears punctate and co-localises majorly with mitochondrial, 82.7% (Pearson’s correlation). Analysis was performed on the presented images. (**B**,**C**) Left panel: anti-HTT (mEM48) antibody (MAB5374) (Alexa Fluor 647), (B: 46Q-VN, C: 97Q-VN). Middle panel: anti-HtrA2/Omi antibody (AF1458) (Alexa Fluor 555). Right panel: merge of HTT and mitochondrial signals. Nuclei were stained with Hoechst 33342. Scale bar = 8 µm. Anti-HTT signal co-localises with the mitochondrial signal, but the presence of aggregates (bright inclusions) makes images unquantifiable. HTT-VN and mitochondrial signals are presented with red and green signals for Alexa Fluor 647 and 555, respectively.

The co-localisation of interaction partners within the cell is crucial for protein–protein interactions to occur. Thus, confocal microscopy studies were conducted to elucidate the cellular localisation of the HTT-VN constructs. We used two parallel immunolabelling approaches to investigate HTT19Q-VN localisation: either anti-HTT (mEM48) + the mitochondrial marker HtrA2 (Figure 5) or anti-GFP + the mitochondrial antibody MAB1273 (Figure S8). Surprisingly, HTT19Q-VN was found to be almost exclusively localised to mitochondria (Figure 5A and Figure S8). Pearson’s correlation between the red (HTT19Q-VN) and the green (anti-HtrA2) signals in the analysed image was very high (Figure 5A), showing 82.7% co-localisation with the mitochondrial marker. When 10 further optical z-sections of different images were analysed, an average co-localisation of 79.7% ± 1.6 was found. Similar mitochondrial localisation was observed with the anti-GFP + MAB1273 combination (72.9% Pearson’s correlation) (Figure S8). Cells transfected with the HTT46Q-VN construct, similar to HTT19Q-VN, exhibited predominantly mitochondrial localisation (Figure 5B and Figure S9A) and the presence of some HTT inclusions (Figure S9B). HTT97Q-VN transfected cells had a large number of HTT inclusions, though some signals still co-localised to mitochondria (Figure 5C and Figure S9C,D).

**Figure 6:**
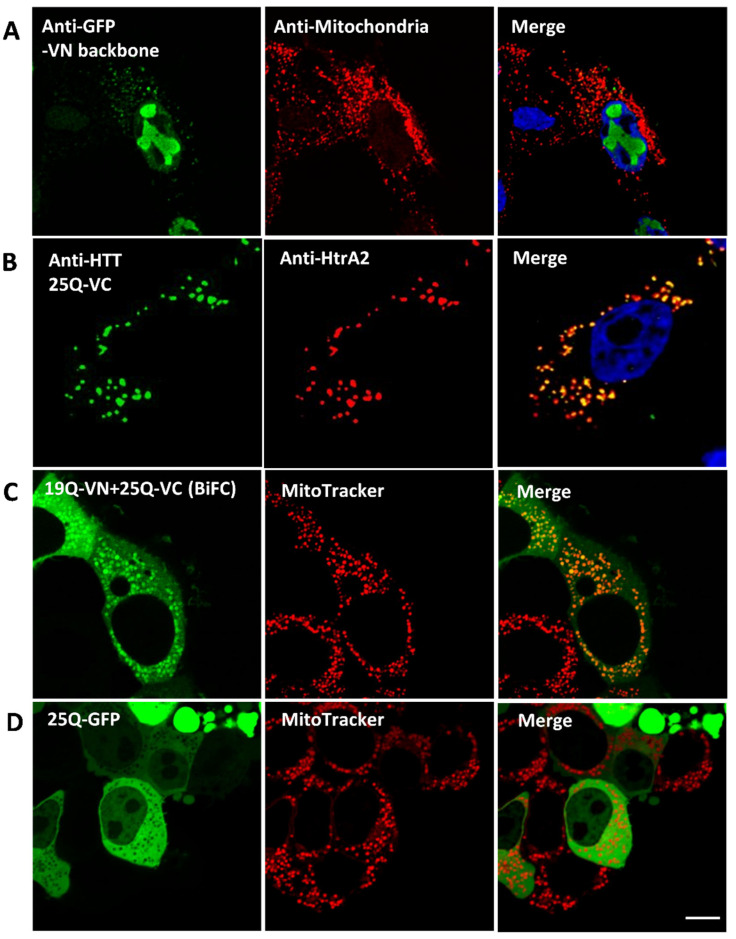
**Fluorescence tag effect on wild type HTT subcellular localisation in fixed HEK293T cells transfected with various constructs for 48 h.** (**A**) N-terminal half of Venus (VN) localisation in dual immunolabelled HEK293T cells. Left panel: anti-GFP (ab6556) (Alexa Fluor 555). Middle panel: anti-mitochondria antibody (MAB1273) (Alexa Flour 647). Right panel: merge of VN and mitochondrial signals, nuclei stained with Hoechst 33342. VN localises mostly in the nucleus, with minor punctate staining in the cytosol which co-localises with mitochondria. (**B**) Cells expressing 25Q-VC were double immunolabelled as follows: Left panel: anti-HTT (mEM48) antibody (MAB5374) (Alexa Fluor 647). Middle panel: anti-HtrA2/Omi antibody (AF1458) (Alexa Fluor 555). Right panel: merge of HTT and HtrA2 signals; nuclei were stained with Hoechst 33342. 25Q-VC co-localises with mitochondria. (**C**,**D**) Cells were seeded in ibiTreat dishes and co-transfected for 48 h with 19Q-VN and 25Q-VC (**C**) or 25Q-GFP (**D**). Live cells were stained with MitoTracker Red CMXRox (M-7512) prior to confocal examination. Acquired images were deconvolved. (**C**) Left panel: BiFC signal 19Q-VN and 25Q-VC. Middle panel: MitoTracker signal. Right panel: merge of BiFC and MitoTracker signals. BiFC signal of WT HTT is mitochondrial (indicated by the co-localisation with MitoTracker) as well as cytosolic. (**D**) Left panel: GFP signal showing the fused 25Q localisation. Middle panel: MitoTracker signal. Right panel: merge of the 25Q-GFP and MitoTracker signals. Scale bar = 8 µm. 25Q-GFP is expressed in the cytosol, with complete exclusion of mitochondrial localisation.

To clarify the effect of the fluorescent tag on HTT cellular localisation, we analysed cells expressing different constructs as controls. We found that (1) expression of the VN fragment on its own led to predominantly nuclear localisation (possibly in the nucleoli) (Figure 6A); (2) expression of WT HTT fused to the C-terminus of Venus (HTT25Q-VC) clearly showed mitochondrial localization (Figure 6B), similar to the results with HTT19Q-VN (Figure 5A); (3) expression of the WT HTT BiFC pair (HTT19Q-VN and HTT25Q-VC) in living cells yielded both mitochondrial and cytosolic signals (Figure 6C); and (4) expression of WT HTT (25Q) fused to full length GFP was predominantly cytosolic and did not co-localise with the mitochondrial marker (Figure 6D). Together, these studies indicate that half Venus tags fused to WT HTT enhance localisation to mitochondria, and thus provide a useful tool for exploring mitochondrial HTT interactions/function. 

### 3.4. KMO and HTT Co-Localise at the Outer Mitochondrial Membrane 

**Figure 7:**
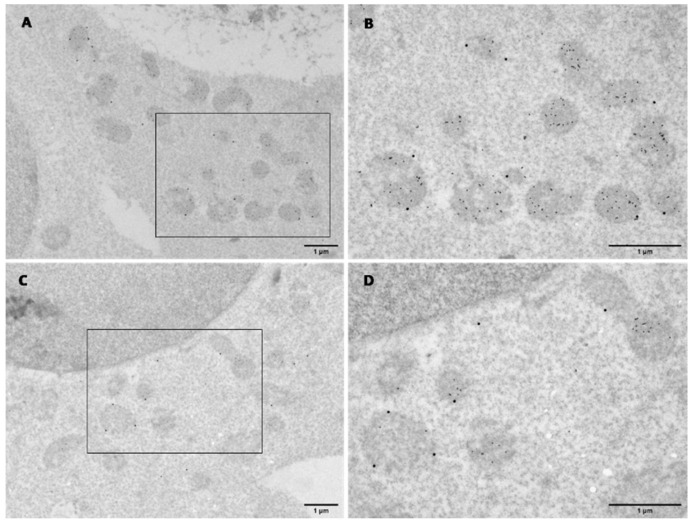
**Electron micrographs of dual immunogold labelling in transfected HEK293T cells for 48 h.** Cells were co-transfected and co-probed with anti-KMO antibody (10698-1-AP) and anti-HTT (mEM48) antibody (MAB5374), followed by 30 and 15 nm gold conjugate secondary antibodies, respectively. (**A**,**C**) Overview of dual labelling of flKMO-CC and 19Q-VN, respectively. Scale bar = 1 µm. (**B**,**D**) Zoomed view of the regions indicated by the black box in (**A**) and (**C**), respectively. Mitochondria are all intensely labelled with HTT (15 nm particles), and some particles are seen in the cytoplasm. flKMO-CC labelling is seen on the outer membrane of some HTT-labelled mitochondria (30 nm particles). Scale bar = 1 µm.

Finally, to extend our findings to the ultrastructural level, we used transmission electron microscopy (TEM). HEK293T cells were co-transfected with flKMO-CC + HTT19Q-VN constructs, followed by immunolabelling with anti-KMO and anti-HTT (mEM48) antibodies Figure 7 and Figure S10). TEM results showed stronger HTT19Q-VN labelling than flKMO-CC, indicated by the presence of 15 nm particles, which were predominantly mitochondrial. flKMO-CC labelling with 30 nm particles was localized to the outer membrane of some mitochondria (Figure 7A,B). Interestingly, these results not only confirmed the presence of both flKMO-CC and 19Q-VN in the same mitochondrion, but also revealed that particles were very close to one another, supporting the likelihood that these two proteins interact physically (Figure 7B). These data for the first time confirm the proposed localisation of KMO by ultrastructural analysis, extending previous studies that detected pig liver KMO at the outer mitochondrial membrane [29,30].

## 4. Discussion

Here we have determined that HTT localised to mitochondria can physically interact with KMO at the outer mitochondrial membrane. Our primary tool for exploring these interactions in living cells was the BiFC assay. CC and VN tags were fused to the two proteins being studied, flKMO and HTT exon 1, respectively. Typically, when constructing BiFC plasmids, a split fluorescent protein such as GFP is used. However, as discovered by Shyu et al. [38], BiFC complementation efficiency is enhanced when using other combinations of fluorescent protein fragments derived from Venus and Cerulean. This approach permitted the visualisation and subcellular localisation of interactions between HTT and KMO in living cells, which were disrupted upon polyQ expansion in HTT. These interactions were confirmed biochemically via co-immunoprecipitation using MYC-tagged constructs. While overexpression of the candidate proteins due to strong promoters relative to endogenous levels of the respective proteins could be a confounding factor, the RFP-Trap pulldown of flKMO-RFP detected an interaction with endogenous HTT (Figure 3F), suggesting that the KMO-HTT interactions observed are not solely due to overexpression of the constructs. Furthermore, and in line with a previous microscopic analysis showing the mitochondrial localisation of KMO [29], we demonstrated by TEM that HTT and KMO co-localised at the outer mitochondrial membrane. Notably, this is the first ultrastructural analysis of KMO, confirming and extending previous studies employing sub-fractionation of mitochondria and showing that KMO localises to the outer membrane of the organelle [30]. 

Several studies have provided evidence of HTT localisation to mitochondria. Electron microscopy studies by Panvo et al. [39] showed that an N-terminal mutant HTT fragment binds to the mitochondrial membrane of neurons derived from YAC72 mice, but not WT YAC18 mice. Using quantitative electron microscopic analysis, Gutekunst et al. [24] found that about 10% of HTT normally associates with mitochondria in the rat brain. In addition, sub-fractionation of mitochondria from a clonal striatal cell line revealed that both WT and mutant full length HTT were associated with the outer mitochondrial membrane [23]. Kegel et al. [40,41,42] investigated the relationship between HTT and membrane phospholipids and observed that both WT and mutant full length HTT associated specifically with cardiolipin (a phospholipid specific to the inner mitochondrial membrane) [41]; moreover, the region 171–287 aa was required for maximal HTT association with cellular membranes [41,42]. More recently, as noted above, HTT has been linked to mitochondrial protein import, with direct interactions detected with TIM23, which is localised to the inner mitochondrial membrane [25,26,27]. Together, these studies highlight that at least a subset of HTT species localises to mitochondria and is therefore available for interactions with KMO. 

The mitochondrial localisation of the HTT-KMO BiFC complexes additionally provides insight into the HTT species that interact with KMO. The absence of fluorescently visible HTT inclusions when flKMO-CC and HTT97Q-VN were co-transfected indicates that flKMO only interacts with soluble HTT localised to mitochondria and is not sequestered into HTT aggregates. This may also explain the inverse relationship between this interaction and polyQ length. In other words, increased polyQ length potentiates aggregation, and consequently less soluble HTT protein is available to interact with flKMO at the outer mitochondrial membrane. As a consequence, a lower fraction of HTT may be accessible at the mitochondria due to HTT being sequestered into cytosolic aggregates.

Recent studies have linked KMO to mitochondrial function and cellular bioenergetics. Specifically, overexpression of KMO in HEK293 cells was found to potentiate production of ROS and increase mitochondrial depolarisation [1]. Interestingly, while mitochondrial respiration analysis of these cells found most parameters unchanged (basal oxygen consumption rate, proton leak, coupling efficiency), spare respiratory capacity was significantly decreased—indicating that respiration was closer to maximal capacity. A separate study demonstrated that overexpression of KMO in HEK293 cells protects against 3-HK toxicity [43]. Complementing these studies, work in our laboratory using *Drosophila melanogaster* revealed a strong link between KMO and mitochondrial dynamics [44]. Indeed, KMO-deficient fruit flies exhibit elongated mitochondria and impaired mitochondrial respiration. Notably, the gene encoding KMO (*cinnabar*) genetically interacts with several genes involved in mitochondrial fission/fusion and mitophagy (*Pink1*, *parkin*, *Drp1*), suggesting a role for KMO in mitochondrial dynamics and maintenance. 

In summary, the present study demonstrates that KMO and HTT can physically interact at the outer mitochondrial membrane, and that this interaction is disrupted by polyQ expansion, which reduces the pool of soluble HTT available at the mitochondria. While we do not see effects on KMO enzymatic activity due to interactions with HTT, we have previously observed that KMO-dependent effects on mitochondrial physiology are not linked to its role in KP metabolism [44]. It is therefore conceivable that interactions with HTT may modulate these KMO-dependent mitochondrial functions, indicating the existence of heretofore unrecognized cellular role(s) of KMO. Future studies will be required to clarify these aspects of KMO-HTT biology in greater detail and to elucidate their significance for HD pathology. 

## Data Availability

Not applicable.

## Supplementary Materials

The following supporting information can be downloaded at: https://www.mdpi.com/article/10.3390/biomedicines10092294/s1: Figures S1–S10 and Tables S1 and S2.
