# Supplementary material for: Kynurenine 3-Monooxygenase Interacts with Huntingtin at the Outer Mitochondrial Membrane
Source: Biomedicines. 2022 Sep 15;10(9):2294. doi: 10.3390/biomedicines10092294 (PMC9496550; doi:10.3390/biomedicines10092294)
Supplement: Supplementary file 1 [file biomedicines-10-02294-s001.zip › biomedicines-1914942-supplementary.pdf]

Supplementary Information

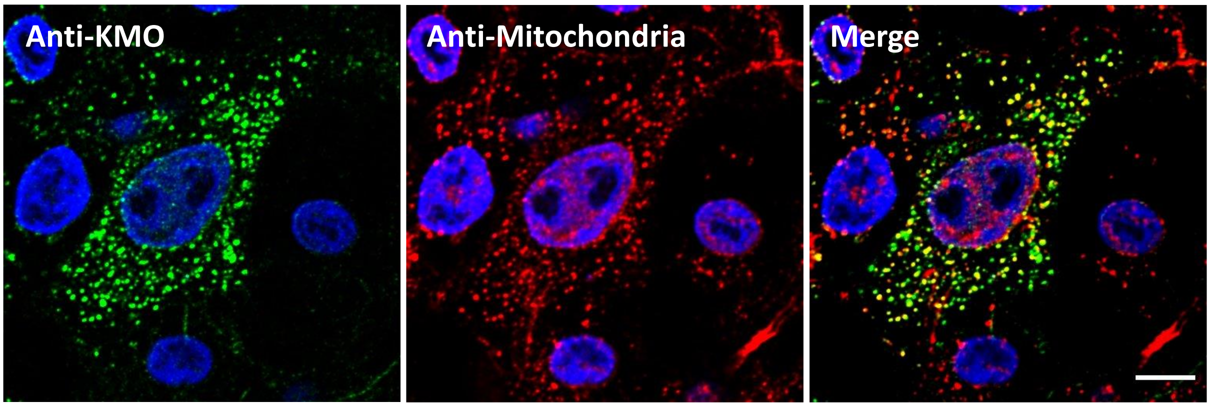

Figure S1. Subcellular localisation of flKMO-CC in fixed HEK293T cells. Images were acquired using confocal microscopy and deconvolved. Anti-KMO antibody (10698-1-AP) immunolabelling of flKMO-CC (Alexa Fluor 555) (left panel). Anti-mitochondria (MAB1273) immunolabelling (Alexa Fluor 647). Green and red were used to represent Alexa Fluor 555 and 647 respectively to illustrate the overlapping (in yellow) as in the right merge panel. Nuclei were stained with Hoechst 33342. Scale bar = 8 µm.

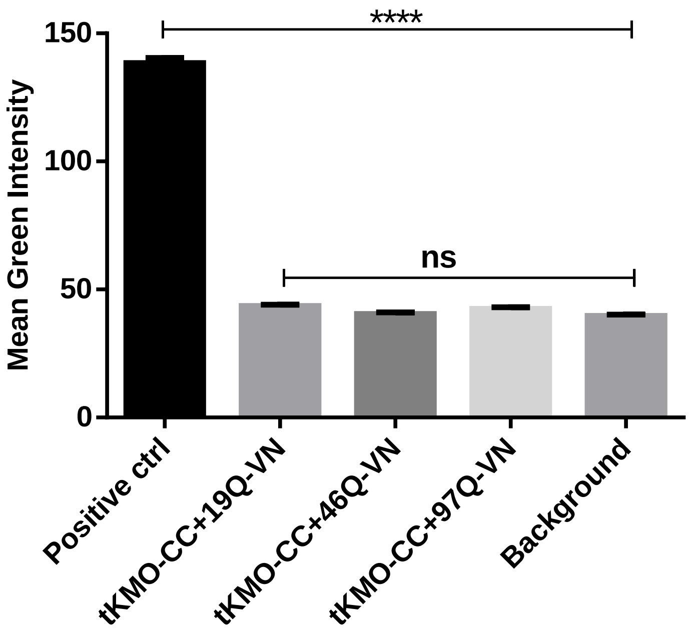

Figure S2. tKMO-CC and HTT BiFC in living HEK293T cells 48 h post transfection. Cells were transfected with 0.16 µg of each plasmid as well as 0.08 µg of RFP. Mean green intensity of different polyQ length HTT-VN constructs co-transfected with tKMO-CC: no interaction was seen in any of the conditions. ****$P < 0.0001$ and ns = not significant, for one-way ANOVA, followed by Tukey's multiple comparison tests. Data are expressed as mean per well ± SEM. The number of analysed cells ranged from 2500 to 4000 cells per well per condition. Background = flKMO-CC + VN-backbone; positive control = DJ-1-GN + DJ-1-CC.

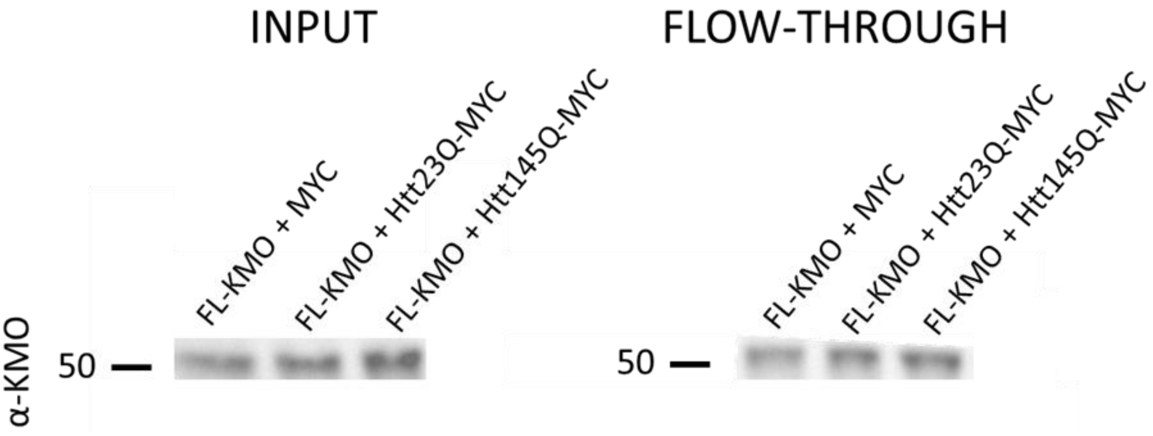

Figure S3. Input and flow-through blots from MYC-Trap immunoprecipitation. HEK293T cells were transfected with untagged FL-KMO with either MYC, HTT23Q-MYC or HTT145Q-MYC constructs. Protein crosslinking was performed using DSP and MYC-Trap was used for pulling down HTT constructs MYC tagged (corresponding IP is shown in Figure 3E in the main text).

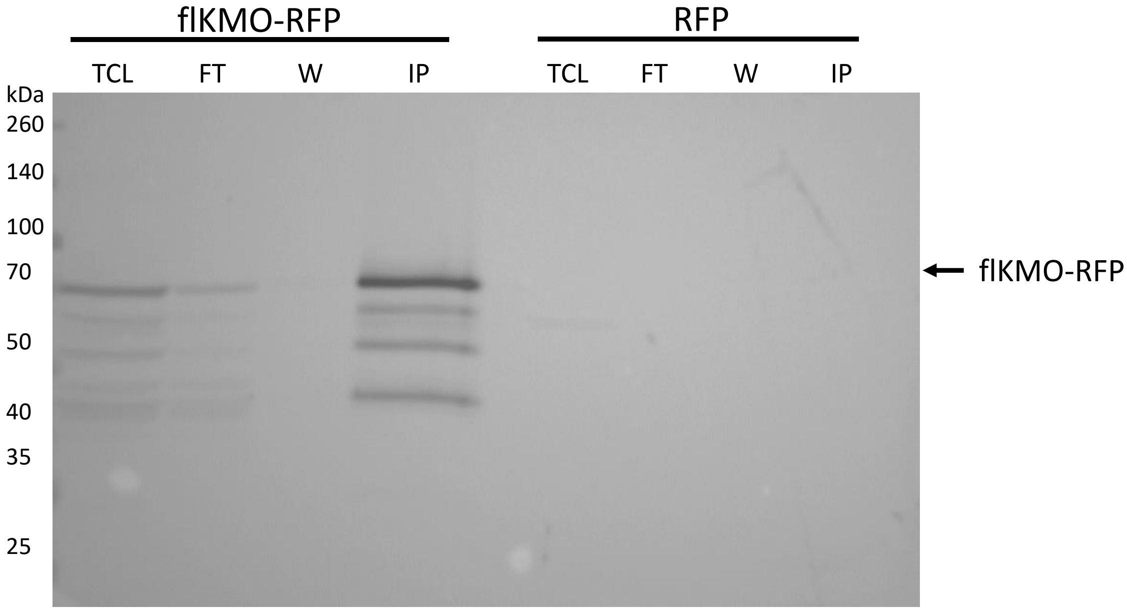

Figure S4. Immunoblot analysis of RFP-Trap agarose pull-down of flKMO-RFP from HEK293T cell lysates. HEK293T cells were transfected with pcDNA3.1-flKMO-RFP or pcDNA3.1-RFP and incubated for 48h to allow expression. flKMO-RFP was pulled down from cell lysates using RFP-Trap agarose and examined by immunoblot analysis using an anti-KMO antibody (MAB8050). TCL = total cell lysate, FT = flow through, W = wash and IP = immunoprecipitation.

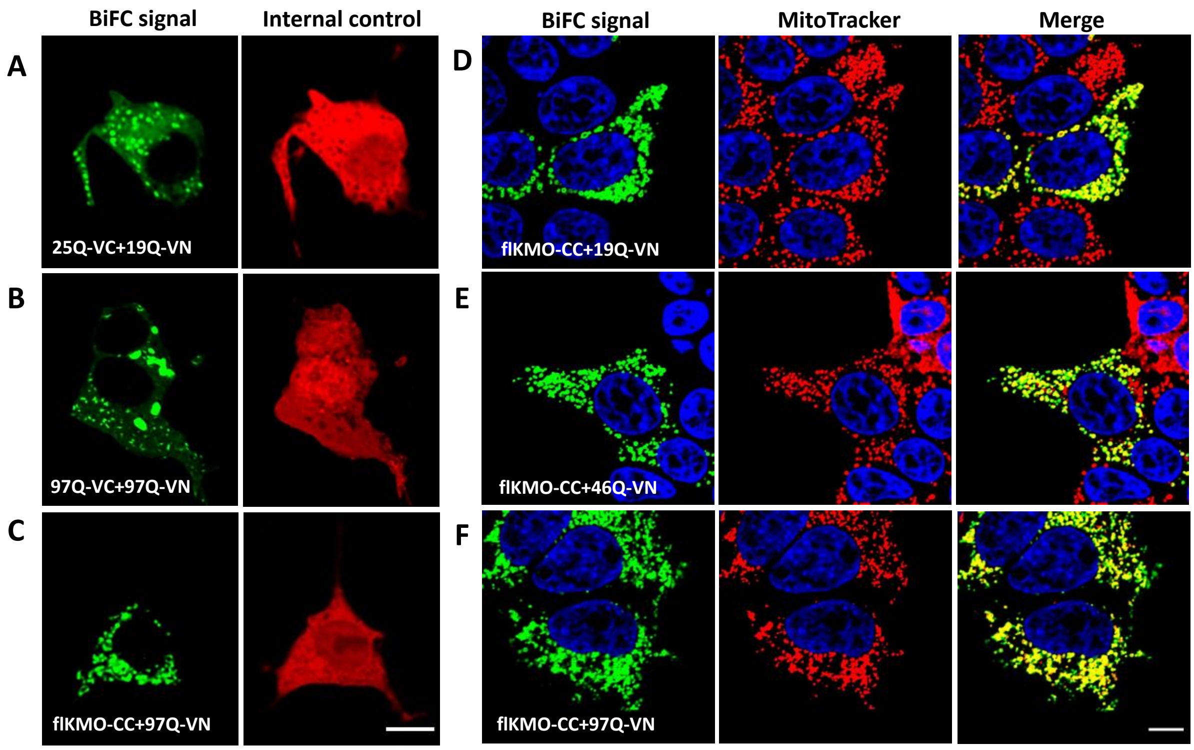

Figure S5. Cellular localisation of BiFC complexes in fixed HEK293T cells, using confocal microscopy. Cells were co-transfected with BiFC constructs for 48 h. A, B and C) Visualising HTT aggregates using BiFC system. BiFC signal = left image, and internal control RFP signal = right image) in each panel. A) Cells were transfected with 19Q-VN, 25Q-VC and RFP, the BiFC signal is both cytosolic and punctate. B) Cells were transfected with 97Q pair and RFP, the BiFC signal seen is generally cytosolic with presence of HTT inclusions. C) Cells were transfected with flKMO-CC, 97Q-VN and RFP; the BiFC signal is mainly mitochondrial as suggested by the dotted appearance of the signal. Scale bar = 8 µm. D, E and F) Localisation of KMO-BiFC complexes. Left panels BiFC signal of: D) flKMO-CC and HTT19Q-VN, E) flKMO-CC and HTT46Q-VN, and F) flKMO-CC and HTT97Q-VN. Second column of panels (D, E and F): mitochondria stained with MitoTracker Red CMXRox (M-7512). Third column of panels (D, E and F): merge of the BiFC signal and the MitoTracker signal. Nuclei were stained with Hoechst 33342. Scale bar = 8 µm. BiFC signal in panels D, E and F presents dotted structures that co-localise with the MitoTracker signal, as seen in the merge images in the right panels of D, E and F.

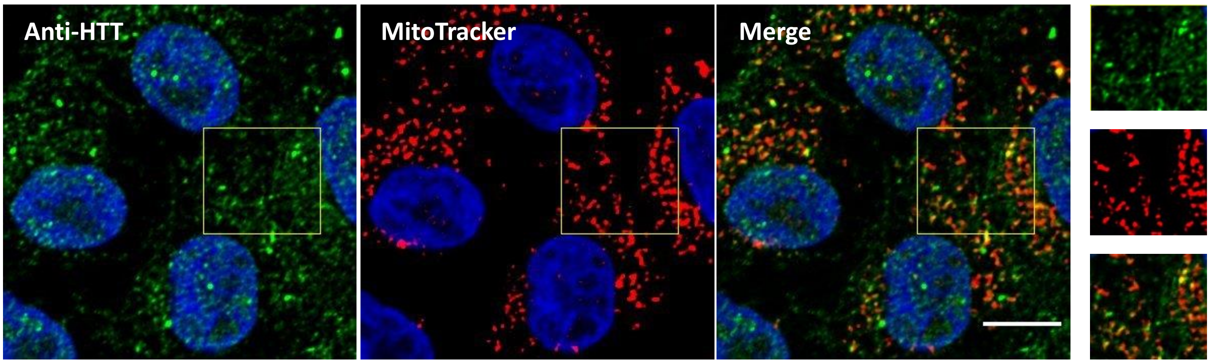

Figure S6. Co-localisation analysis of endogenous HTT with mitochondria on deconvolved confocal optical z-sections of HEK293T cells. Left panel: HTT immunolabelling using anti-HTT (4C8) antibody (MAB2166) (Alexa Fluor 488). Middle panel: MitoTracker Red CMXRos (M-7512) staining. Right panel: merge image of the HTT and mitochondrial signals. Nuclei were stained with Hoechst 33342. Scale bar = 8 µm. Zoomed-in views are presented to the right of the panels. HTT signal is punctate and co-localises partially with the mitochondrial signal.

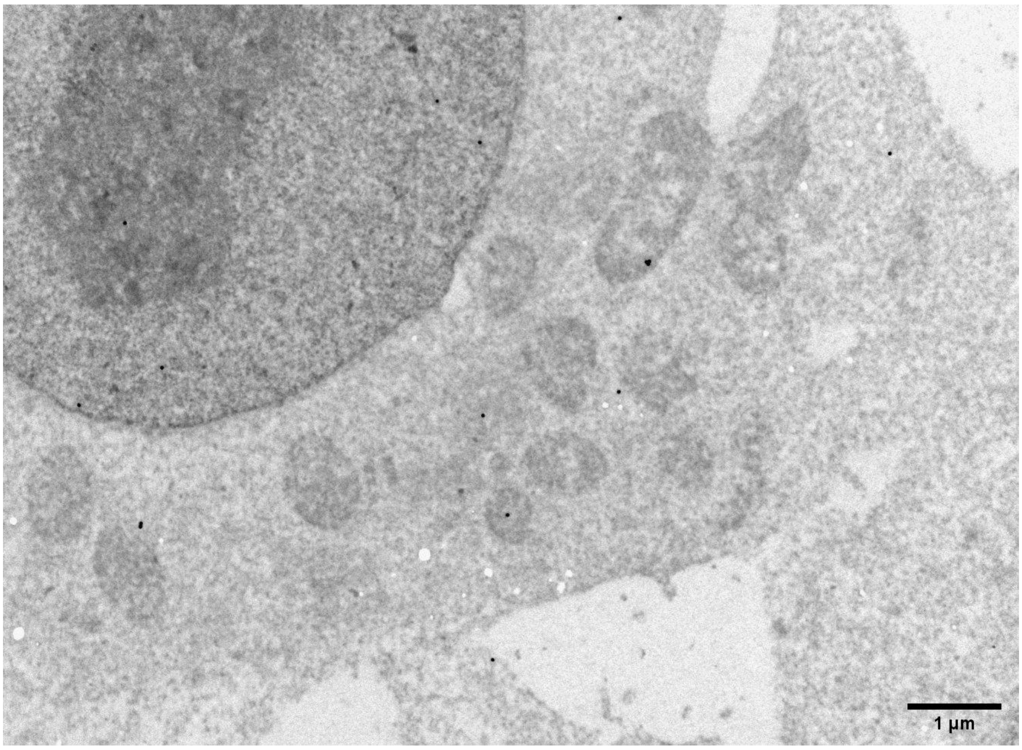

Figure S7. Electron micrograph of endogenous HTT in HEK293T cells. HTT immunogold labelling was using anti-HTT, 4C8 antibody (MAB2166). Scale bar = 1 µm.

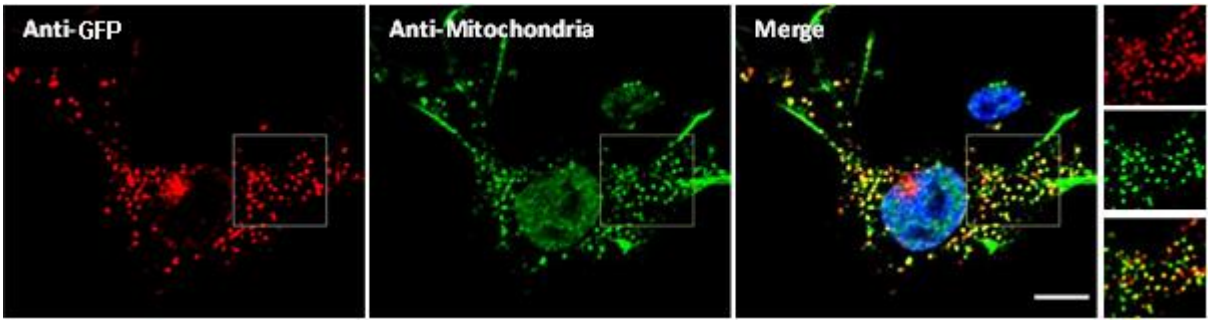

Figure S8. Co-localisation analysis of 19Q-VN and mitochondrial fluorescent signals on deconvolved confocal optical z-sections, using JACoP plugin in ImageJ. HEK293T cells were transfected with 19Q-VN, and after 48 h cells were fixed and immunolabelled. Left panel, 19Q-VN immunolabelling of anti-GFP antibody (ab6556) (Alexa Fluor 555). Middle panel, mitochondrial immunolabelling of anti-mitochondria antibody (MAB1273) (Alexa Flour 647). Right panels: merge image of the HTT and mitochondrial signals. Nuclei were stained with Hoechst 33342. Scale bar = 8 µm. Co-localisation analysis were carried out on the regions of interest indicated on the images; and a zoomed-in is presented on the right of the merge panel. 19Q-VN signal co-localises with mitochondria, 72.9% (Pearson's correlation). Analysis was done on the presented images. 19Q-VN and mitochondrial signals are presented with green and red signals for Alexa Fluor 555 and 647, respectively.

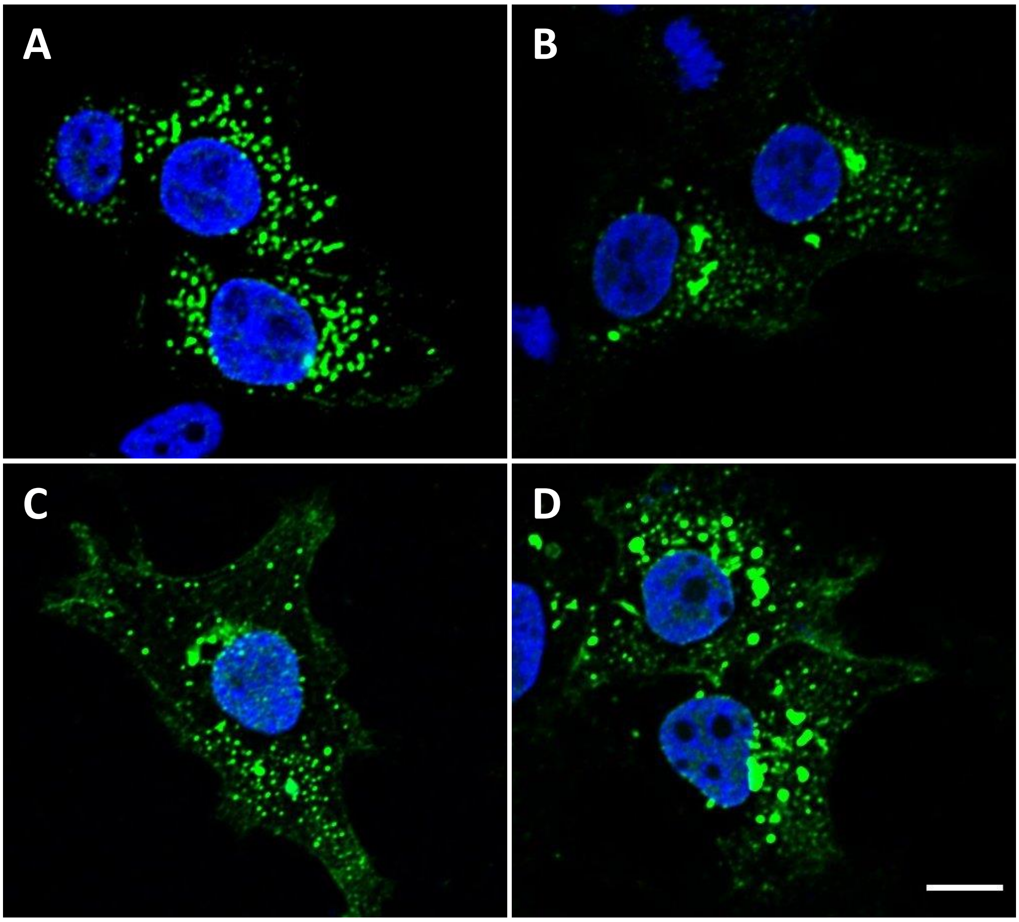

Figure S9. Deconvolved confocal images of mHTT in HEK293T cells. Cells were transfected with either 46Q-VN (A and B) or 97Q-VN (C and D) for 48 h. Cells were then fixed and immunolabelled with anti-GFP anti-body (ab6556) (Alexa Fluor 647). Nuclei were stained with Hoechst 33342. Scale bar = 8 µm. Clearly, cells in B and D (46Q-VN and 97Q-VN respectively) showed more inclusions than A (46Q-VN) and C (97Q-VN).

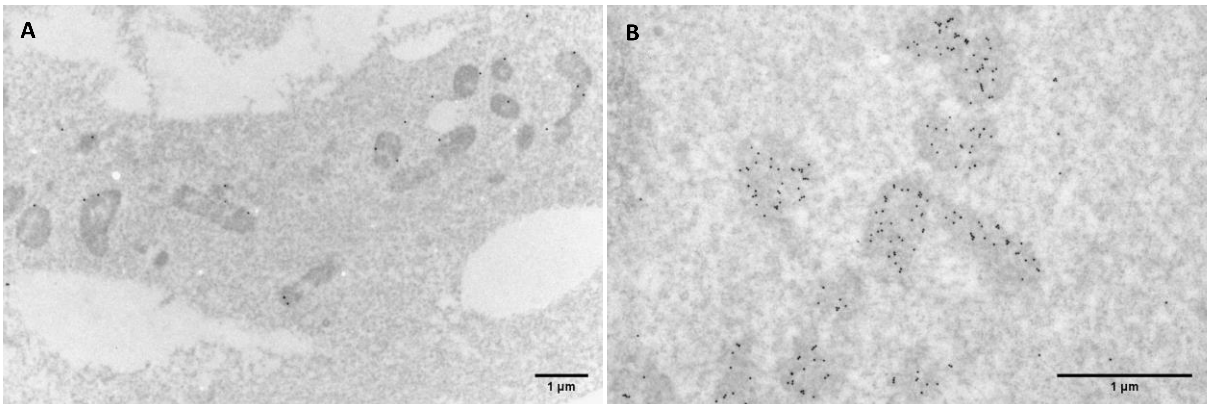

Figure S10. Electron micrographs of transfected HEK293T cells. Cells were transfected for 48 h with either flKMO-CC or HTT19Q-VN. Immunogold labelling shows sub-cellular localisation of the transfected proteins. The darkened structures are the mitochondria. A) flKMO-CC immunogold labelling is clearly on the outer surface of mitochondria; anti-KMO antibody (10698-1-AP). Scale bar = 1 µm. B) Gold particles labelling HTT19Q-VN were heavily mitochondrial, with few others in the cytosol; anti-HTT, mEM48 antibody (MAB5374). Scale bar = 1 µm.

Table S1 PCR primers.

Primer	Sequence	Application
KMO-RFP_Fw	GATC**GGTACC**GCCACCATGGACTCATCTGTCATTCAAAG	Forward primer for KMO introducing *KpnI* site
KMO-RFP_Rev	GATC**GGTACC**GCCCTGCTAATGAGATTGGAAATTTG	Reverse primer for KMO introducing *KpnI* site
tKMO-RFP_Rev	GATC**GGTACC**CTTTTTTGCCAATGCCAACGCTGCAC	Reverse primer for tKMO introducing *KpnI* site
KMOBiFC_Fw	GATC**GCTAGC**GCCACCATGGACTCATCTGTCATTCAAAG	Forward primer for KMO introducing *NheI* site
KMOBiFC_Rev	GATC**GTCGAC**GGAGCCTCCCCCGCCGGAGCCTCCCCCGCCCC TGCTAATGAGATTGGAAATTTG	Reverse primer for KMO introducing *SalI* site and $(GGGGS)_2$ linker
tKMOBiFC_Rev	GATC**GTCGAC**GGAGCCTCCCCCGCCGGAGCCTCCCCCGCCTT TTTGCCAATGCCAACGCTGCAC	Reverse primer for tKMO introducing *SalI* site and $(GGGGS)_2$ linker
flKMO_Fw	GATCGGTACCGCCACCATGGACTCATCTGTCATTCAAAG	Forward primer for flKMO introducing *KpnI* site
flKMO_Rev	GATCCTGCAGTCACCTGCTAATGAGATTGGAAATTTGTTC	Reverse primer for flKMO introducing *PstI* site
MYC_Fw	CGTAGGTACCGCCACCATGGAACAAAAACTCATCTCAGAAGA GGATCTGAATATGCATACCGGTCATCATCACCATCACCATTG ACTGCAGCGTAT	Forward primer for MYC
MYC_Rev	ATACGCTGCAGTCAATGGTGATGGTGATGATGACCGGTATGC ATATTCAGATCCTCTTCTGAGATGAGTTTTTGTTCCATGGTG GCGGTACCTACG	Reverse primer for MYC

Table S2 List of primary antibodies and dilutions.

Catalogue #	Antigen	Host species/Isotype	Supplier	Application	Dilution
ab6556	GFP	Rabbit polyclonal	Abcam	IB	1:5000
				ICC	1:1000
10698-1-AP<br>Lot # 00000888	Human KMO	Rabbit polyclonal	Proteintech	IB	1:1000
				ICC	1:100
				TEM	1:10
MAB8050	anti-KMO antibody	Rabbit monoclonal	R&D systems	IB	1:1000
sc-8035	α-Tubulin	Mouse monoclonal	Santa Cruz Biotechnology	IB	1:1000
sc-32233	Anti-GAPDH	Mouse Monoclonal	Santa Cruz Biotechnology	IB	1:1000
AF1458	HtrA2	Rabbit polyclonal	R&D systems	ICC	1:400
MAB1458	HtrA2	Mouse monoclonal	R&D systems	ICC	1:200
MAB1273	Anti-Mitochondria	Mouse Monoclonal	Chemicon	ICC	1:200
MAB5374	Anti-HTT (mEM48)	Mouse Monoclonal	Chemicon	IB	1:2000
				ICC	1:1000
				TEM	1:250
MAB2166	Anti-HTT-FL (4C8)	Mouse Monoclonal	Chemicon	IB	1:1000 or 1:2000
				ICC	1:1000
				TEM	1:20

IB = Immunoblot, ICC = Immunocytochemistry, TEM = Transmission electron microscopy
